# What Is the Most Effective Way of Increasing the Bioavailability of Dietary Long Chain Omega-3 Fatty Acids—Daily *vs.* Weekly Administration of Fish Oil?

**DOI:** 10.3390/nu7075241

**Published:** 2015-07-10

**Authors:** Samaneh Ghasemifard, Andrew J. Sinclair, Gunveen Kaur, Paul Lewandowski, Giovanni M. Turchini

**Affiliations:** 1 School of Medicine, Deakin University, Geelong 3220, Australia; E-Mails: sghasemi@deakin.edu.au (S.G.); andrew.sinclair@deakin.edu.au (A.J.S.); paul.lewandowski@deakin.edu.au (P.L.); 2 Centre for Physical Activity and Nutrition Research (CPAN), School of Exercise and Nutrition Sciences, Deakin University, Burwood 3125, Australia; E-Mail: Gunveen.Kaur@deakin.edu.au; 3 School of Life and Environmental Sciences, Deakin University, Warrnambool 3280, Australia

**Keywords:** bioavailability, EPA, DHA, DPA, metabolic fate, tissue deposition, frequency of intake

## Abstract

The recommendations on the intake of long chain omega-3 polyunsaturated fatty acids (*n*-3 LC-PUFA) vary from eating oily fish (“once to twice per week”) to consuming specified daily amounts of eicosapentaenoic acid (EPA) and docosahexaenoic acid (DHA) (“250–500 mg per day”). It is not known if there is a difference in the uptake/bioavailability between regular daily consumption of supplements*vs.* consuming fish once or twice per week. In this study, the bioavailability of a daily dose of *n*-3 LC-PUFA (Constant treatment), representing supplements, *vs.* a large weekly dose of *n*-3 LC-PUFA (Spike treatment), representing consuming once or twice per week, was assessed. Six-week old healthy male Sprague-Dawley rats were fed either a Constant treatment, a Spike treatment or Control treatment (no *n*-3 LC-PUFA), for six weeks. The whole body, tissues and faeces were analysed for fatty acid content. The results showed that the major metabolic fate of the *n*-3 LC-PUFA (EPA+docosapentaenoic acid (DPA) + DHA) was towards catabolism (β-oxidation) accounting for over 70% of total dietary intake, whereas deposition accounted less than 25% of total dietary intake. It was found that significantly more *n*-3 LC-PUFA were β-oxidised when originating from the Constant treatment (84% of dose), compared with the Spike treatment (75% of dose). Conversely, it was found that significantly more *n*-3 LC-PUFA were deposited when originating from the Spike treatment (23% of dose), than from the Constant treatment (15% of dose). These unexpected findings show that a large dose of *n*-3 LC-PUFA once per week is more effective in increasing whole body *n*-3 LC-PUFA content in rats compared with a smaller dose delivered daily.

## 1. Introduction

A vast body of literature exists on the effect of long chain omega-3 polyunsaturated fatty acids (*n*-3 LC-PUFA) in the areas of infant development [1], cardiovascular disease, platelet aggregation [2], cancer [3], dementia, Alzheimer’s disease, depression [4,5], and inflammation [6,7].

Recommendations for eicosapentaenoic acid (EPA; 20:5n-3) plus docosahexaenoic acid (DHA; 22:6n-3) intake have been put forth by several organizations globally, with ranges from 250 mg to 500 mg per day for adults with an additional 200 mg of DHA per day for pregnant and lactating women [8]. The recommendation for patients with coronary heart disease generally is 1 g EPA plus DHA per day, and for patients with high triglycerides the recommendations range from 1.2 to 4 g EPA plus DHA per day [8]. Some organisations, including the American Heart Association and Australian National Health and Medical Research Council, recommend foods (oily fish) once to twice per week [9], whereas others like the National Heart Foundation of Australia deal with nutrients daily based on daily intake (250–500 mg per day of EPA plus DHA) [10]. Accordingly, despite the actual quantity of recommended intake, it appears to be important to understand what feeding strategy, such as a constant daily dose or large doses fewer times per week, is more efficient to fulfil dietary requirements of EPA and DHA.

There are two human studies which have compared a constant daily dose of *n*-3 LC-PUFA *vs.* twice weekly doses of *n*-3 LC-PUFA [11,12]. Harris *et al.* [11] compared the bioavailability of EPA and DHA from twice weekly consumption of oily fish (3.40 g/week EPA + DHA) with daily fish-oil capsule supplementation (3.37 g/week EPA + DHA) in a 16 week study in 23 women; they found that there were no treatment differences in the EPA and DHA content of red blood cell membranes or plasma phospholipids. Browning *et al.* [12] compared the bioavailability of EPA and DHA from fish oil capsules (twice per week) with daily fish oil consumption. Male and female participants (*n* = 65) were given capsules (containing 6.54 g of EPA and DHA per week) either twice weekly or daily for one year. They found that there was no difference between treatments in the plasma phosphatidylcholine level of EPA and DHA, but that there were significantly higher levels of EPA and DHA in platelets and higher levels of EPA in mononuclear cells. Both studies used blood (plasma or red cell) levels of *n*-3 LC-PUFA as a proxy for bioavailability, which as discussed previously has significant limitations [13].

The aim of the present study was to compare the bioavailability and efficiency (as metabolic fate) of the same amount of dietary *n*-3 LC-PUFA administered either as a constant daily dose (Constant treatment)*vs.* a single weekly dose (Spike treatment). It was hypothesized that a single weekly dose of *n*-3 LC-PUFA would not be as bioavailable as a continuous daily dose of *n*-3 LC-PUFA.

## 2. Materials and Methods

### 2.1. Diet and Study Design

This study was performed following the Australian code for the care and use of animals for scientific purposes and approved by the Deakin University Animal Welfare Committee (G29-2012).

Forty eight 6-week old, sexually mature, healthy male Sprague-Dawley rats were purchased from Animal Resources Centre, Western Australia. Rats were housed in pairs (2 rats per cage; 24 cages in total) and acclimatised for a week on *ad libitum* normal chow diet. The 24 cages of rats were randomly divided into four groups of six cages each. One group (six cages) was sacrificed via CO_2_ overdose at day 0 for the baseline data. The other three groups (six cages each) were randomly allocated to three different dietary treatments (feeding regimes), named “Control”, “Constant” and “Spike”. To achieve these three different dietary treatments, three specifically formulated diets were designed and manufactured to be iso-proteic, iso-lipidic (10% fat by weight, Speciality Feeds, Western Australia), and named: “No *n*-3 LC-PUFA diet”, “Constant diet” and “Spike diet”. The “No *n*-3 LC-PUFA diet” was formulated to contain no fish oil, and thus no *n*-3 LC-PUFA; the “Constant diet” was formulated to contain 0.7% of fish oil (mixed fish oils), and the “Spike diet” was formulated to contain a 4.9% of the same fish oil (a 7-fold higher level of *n*-3 LC-PUFA compared with the “Constant diet”) (Table 1). Based on these three experimental diets, three different dietary treatments (feeding regimes) Control, Constant and Spike were implemented. In the “Control” treatment rats were fed only with the “No *n*-3 LC-PUFA diet”, in the “Constant” treatment rats were fed only with the “Constant diet”, and in the “Spike” treatment the rats were fed 6 days/week with the “No *n*-3 LC-PUFA diet”, and one day per week with the “Spike diet”. To ensure equal intake amongst dietary treatments during the experimentation, animals were fed to fixed predetermined ration, which was adjusted weekly relative to body weight, for 6 weeks. To determine the appropriate food ration, a preliminary short trial was implemented over a three-week period, where 15 rats of similar size were fed ad-libitum and total feed consumption was recorded. Then, using a regression equation comparing body weight (BW), the daily ration in grams, was estimated as:

Ration (% BW) = −0.0283BW + 16.6

Therefore, the experimental design for the rats in the Constant and Spike treatments was to consume exactly the same amount of fish oil (and therefore *n*-3 LC-PUFA) over a 7-day period, in order to establish if there was a difference in whole body bioavailability between these two feeding strategies (daily *vs.* weekly).

Throughout the 6-week trial period, the rats were weighed (twice per week), and faeces were collected every day during the last two weeks. After the 6 weeks, the rats were humanely sacrificed via CO_2_ overdose. Six rats from each treatment (one per cage) were used for analysis of whole body lipids and the other six rats (one per cage) were used for the analysis of the individual tissue lipid contents. The collected faeces, whole body and tissues (liver, heart, white gastrocnemius muscle and perirenal adipose tissue) were analysed for fatty acid content.

**Table 1:** Experimental diet formulation, proximate composition and selected fatty acid concentrations.

Composition	No *n*-3 LC-PUFA Diet	Constant Diet	Spike Diet
(a) Diet Formulation and Proximate Composition *			
**Diet formulation (%)**			
Sucrose	10.00	10.00	10.00
Casein (acid)	20.00	20.00	20.00
Starch	37.44	37.44	37.44
Dextrinised starch	13.20	13.20	13.20
Safflower oil	0.05	0.13	0.64
Palm oil	9.88	9.11	4.45
Linseed oil	0.07	0.06	0.01
Fish oil **	0.00	0.70	4.90
**Proximate Composition (mg/g of Diet)**			
Protein	194.00	194.00	194.00
Fibre	84.00	84.00	84.00
Fat	100.00	100.00	100.00
**(b) Fatty Acid Concentration of the Diet After Formulation(mg/g of Diet)**			
16:0	43.00	38.50	26.40
18:0	4.00	3.81	3.10
18:1n-9	28.20	26.90	18.21
18:2n-6	7.71	7.82	7.20
20:4n-6	0.00	0.05	0.27
18:3n-3	0.50	0.50	0.30
18:4n-3	0.00	0.10	0.71
20:5n-3 (EPA)	0.00	0.90	4.50
22:5n-3 (DPA)	0.00	0.12	0.51
22:6n-3 (DHA)	0.00	0.60	2.92

EPA (eicosapentaenoic acid), DHA (docosahexaenoic acid), DPA (docosapentaenoic acid); * Diet formulation and proximate composition reported from data from the feed company; ** Fish oil was a mixture of fish oils used in diet manufacture. The mixture was made at the diet formulators premises before use; *n*-3 LC-PUFA, long chain omega-3 polyunsaturated fatty acids.

### 2.2. Lipid Analysis

The whole body, tissues and faeces samples were homogenized, and the lipids extracted by dichloromethane:methanol (2:1), a modification of the method described by Folch *et al.* [14]. Fatty acids derived from the lipids were methylated using an acid-catalysed trans-methylation [15]. In brief, an aliquot of fatty acids derived from the lipids plus a known amount of internal standard of tricosanoic acid (C23:0 >99%; Nu-Chek Prep Inc., Elysian, MN, USA) were reacted with acetyl chloride/methanol to form fatty acid methyl esters (FAME). The resulting FAME were separated, identified and quantified using an Agilent Technologies 7890A gas chromatography system (Agilent Technologies; Santa Clara, CA, USA) equipped with a BPX70 capillary column (120 m, 0.25 mm internal diameter, 0.25 μm film thickness; SGE Analytical Science, Melbourne, Australia), an Agilent Technologies 7693 autosampler, a split injection system and a flame ionisation detector using established protocols [16].

### 2.3. Lipid and Fatty Acid Apparent Digestibility

Apparent digestibility of total lipid was measured according to Tou *et al.* [17] as ((lipid intake − faecal lipid)/(lipid intake)) × 100. Similarly, apparent digestibility of individual fatty acids was measured using the formula ((fatty acid intake − fatty acid excretion in faeces)/(fatty acid intake)) × 100. Lipid intake was determined as diet consumed per week × % lipid in the diet. Likewise, fatty acid intake was determined as diet consumed per week × % fatty acid in the diet. Faecal lipid and fatty acids were determined as total faeces excreted per week (pooled 7 days faecal samples) × % lipid and % fatty acids in the faeces respectively.

### 2.4. Whole Body Fatty Acid Balance Method

The fatty acid metabolism of rats was determined using the Whole Body Fatty Acid Balance Method, as conceived and described by Turchini *et al.* [18], with subsequent developments [19]. Briefly, the first step of the method required that the net appearance or disappearance of each individual fatty acid be determined by the difference between total fatty acid accumulation (=final fatty acid content-initial fatty acid content) and the net fatty acid intake (=total fatty acid intake-fatty acid excretion in faeces), as initially proposed by Cunnane’s group [20,21,22]. Then, after the transformation of data from gram per animal per the duration of the trial to mol of fatty acid per gram of body weight per day, the subsequent second step involved a series of backwards computations along all the known fatty acid bioconversion pathways, therefore the fate of each individual fatty acid towards bioconversion, β-oxidation or deposition was determined and quantified. Eventually, data relative to apparent *in vivo* enzyme activity could be reported as nmol of enzyme’s product per gram of body weight per day, and the *in vivo* metabolic fate (absorption, β-oxidation, bioconversion and deposition) of each dietary fatty acid could be reported as a % relative to the dietary intake.

### 2.5. Statistical Analysis

All data are reported as mean ± SD (standard deviation) of the six cages per treatment (*n* = 6, *N* = 18). The experimental unit was the cage (with two rats per cage). Significant differences between experimental treatments were tested using one-way analysis of variance (ANOVA), assessing the effects of diets, with an exception of using *t*-test for the *n*-3 LC-PUFA apparent digestibility (as only two treatments were considered). Paired tests were performed with Tukey’s test. Statistical significance was considered for *p* < 0.05. Data analysis was performed with Minitab Statistical Software (Version 16; Minitab Inc., State College, PA, USA).

## 3. Results

### 3.1. Feed and n-3 LC-PUFA Intake

As described in the methods, rats were fed to fixed predetermined ration, which was adjusted weekly relative to body weight, for 6 weeks. Rats in the Control and Spike treatments consumed all the diet provided each week; however, rats in the Constant treatment left some food uneaten, especially in week three, and this was recorded and accounted for (Table 2).

**Table 2:** Actual feed intake over the six weeks of the experiment in the three treatments.

Total Diet Intake (g/rat/6 weeks)	Control	Constant	Spike
No *n*-3 LC-PUFA diet	938 ± 32	-	-
Constant diet	-	931 ± 44	804 ± 25
Spike diet	-	-	134 ± 11

Values are expressed as the mean ± SD of 6 cages per group; *n*-3 LC-PUFA, long chain omega-3 polyunsaturated fatty acids.

An important result that has to be noted and carefully considered was the fatty acid concentration of the experimental diets. Despite the prescribed formulation with 0.7% of fish oil in “Constant diet”, and 4.9% fish oil in “Spike diet” (and fish oil being the only source of dietary *n*-3 LC-PUFA), the resulting manufactured diets had slightly different total amounts of *n*-3 LC-PUFA than expected (Table 1b). The “Constant diet” recorded EPA + DHA + DPA = 1.6 mg/g of diet, and the “Spike diet” recorded EPA + DHA + DPA = 8.0 mg/g of diet (a 5-fold difference, rather than the expected 7-fold difference).

### 3.2. n-3 Fatty Acid Apparent Digestibility

The effect of Constant and Spike treatments on apparent digestibility is shown in Table 3. There were small, but significant differences observed in EPA and DHA apparent digestibility between these two treatments, which was higher in the Constant, compared with the Spike treatment. However, DPA apparent digestibility was similar between Constant and Spike treatments.

**Table 3:** The effect of Constant and Spike treatments on the long chain omega-3 polyunsaturated fatty acids (*n*-3 LC-PUFA) apparent digestibility.

	Constant	Spike	*p*-Value
20:5n-3 (EPA)	99.4 ± 0.0	99.0 ± 0.9	0.021
22:5n-3 (DPA)	96.9 ± 1.0	96.5 ± 0.4	0.753
22:6n-3 (DHA)	98.8 ± 0.5	98.2 ± 0.2	0.042

Values are expressed as the mean ± SD of 6 cages per group; Control treatment not reported as no *n*-3 LC-PUFAwas provided by the diet; EPA, eicosapentaenoic acid; DPA, docosapentaenoic acid; DHA, docosahexaenoic acid.

### 3.3 Growth and Biometrical Parameters

There was no significant difference (*p* = 0.856) in the body weight of rats between the three experimental treatments during the six weeks of the experiment. The average body weights (±SD) of animals in the Control, Constant and Spike treatments were 412 ± 6 g, 413 ± 6 g, and 409 ± 11 g respectively, at the end of the experiment (Table 4).

In regard to the tissue weight, there was no significant difference in the tissue weight of rats between the three dietary treatments at the end of the experiment. In addition, the Hepatosomatic Index and Perirenal adipose-somatic index showed no significant difference in the Constant and Spike treatment compared with the Control (Table 4).

**Table 4:** The effect of Constant and Spike treatments on rat tissue weight and biometric parameters.

Weight (g)	Control	Constant	Spike	*p*-Value
Whole body	412.4 ± 6.2	412.6 ± 6.2	409.3 ± 10.8	0.714
Liver	17.9 ± 2.5	17.3 ± 2.1	17.0 ± 0.8	0.914
Heart	1.5 ± 0.2	1.5 ± 0.1	1.5 ± 0.1	0.547
Perirenal adipose	7.6 ± 2.9	6.8 ± 2.9	8.5 ± 1.9	0.225
Muscle	0.8 ± 0.1	0.7 ± 0.1	0.7 ± 0.1	0.856
HSI*	4.3 ± 0.6	4.2 ± 0.6	4.0 ± 0.3	0.432
PASI**	1.8 ± 0.7	1.7 ± 0.7	2.0 ± 0.4	0.543

Control, Constant and Spike treatments provided 0 mg, 9.2 mg and 8 mg per week of EPA + DPA + DHA, respectively; Values are expressed as the mean ± SD of 6 cages per group; * HSI (Hepatosomatic Index, liver weight/whole body weight × 100); ** PASI (Perirenal adipose-somatic index, Perirenal adipose weight/whole body weight × 100).

### 3.4. Lipid Content of Rat Whole Body and Tissues

Whole body and tissue lipid content (mg/g tissue wet weight) of all rats at the conclusion of the study were significantly higher compared with the baseline data (*p* < 0.001). There was no significant difference in the total lipid content of the whole body or tissues between the three dietary treatments (Table 5).

**Table 5:** Lipid content of tissues of rats fed the different experimental treatments.

Lipid Content	Control	Constant	Spike	*p*-Value
**Whole Body (mg/g Whole Body)**	133.7 ± 17.1	119.1 ± 12.6	111.4 ± 28.8	0.199
**Tissues (mg/g Tissue)**				
Liver	53.3 ± 16.9	51.6 ± 6.4	51.1 ± 8.1	0.942
Heart	29.8 ± 2.6	28.8 ± 0.8	30.7 ± 1.4	0.168
Perirenal adipose	831.2 ± 89.4	893.0 ± 27.4	931.2 ± 67.9	0.640
Muscle	19.5 ± 3.5	20.0 ± 2.8	24.7 ± 8.0	0.209

Values are expressed as the mean ± SD of 6 cages per group.

### 3.5. The Fatty Acid Concentration of the Rat Whole Body and Tissues

As shown in Table 6, the whole body fatty acid concentration for rats that received the Constant treatment was significantly higher for all *n*-3 LC-PUFA compared with rats on the Spike treatment at the end of experiment (*p* < 0.05). In addition, *n*-6 PUFA concentrations were significantly lower in the Spike compared with the Constant treatment at the end of the study period (*p* = 0.003). It should be recalled that rats in the Spike treatment received a relatively smaller amounts of *n*-3 LC-PUFA each week compared with the Constant treatment, because the manufactured diet slightly deviated from expected final fatty acid concentration.

**Table 6:** Selected fatty acid content of whole body (mg/g whole body) and tissues (mg/g tissue) from rats fed different dietary treatments.

	PUFA *n*-6	PUFA *n*-3	EPA	DPA	DHA	EPA + DPA + DHA
**Whole Body** **(mg/g Whole Body)**						
Control	11.23 ± 1.50^b^	0.83 ± 0.12^a^	n.d	0.06 ± 0.01^a^	0.30 ± 0.08^a^	0.37 ± 0.09^a^
Constant	10.88 ± 1.12^b^	1.76 ± 0.08^c^	0.16 ± 0.02^b^	0.22 ± 0.02^c^	0.88 ± 0.06^c^	1.26 ± 0.08^c^
Spike	9.30 ± 1.38^a^	1.49 ± 0.13^b^	0.12 ± 0.02^a^	0.19 ± 0.02^b^	0.75 ± 0.05^b^	1.07 ± 0.07^b^
*p*-Value	0.003	0.001	0.001	0.001	0.001	0.001
**Liver** **(mg/g Liver)**						
Control	7.1 ± 1.7^b^	1.1 ± 0.2^a^	0.03 ± 0.01^a^	0.05 ± 0.01^a^	0.61 ± 0.19^a^	0.68 ± 0.19^a^
Constant	5.7 ± 1.1^a^	2.2 ± 0.3^b^	0.31 ± 0.03^b^	0.14 ± 0.02^b^	1.47 ± 0.32^b^	1.92 ± 0.37^b^
Spike	7.8 ± 1.0^b^	2.6 ± 0.5^b^	0.28 ± 0.08^b^	0.19 ± 0.05^c^	1.72 ± 0.50^b^	2.20 ± 0.56^b^
*p*-Value	0.049	0.001	0.001	0.001	0.001	0.001
**Heart** **(mg/g Heart)**						
Control	6.01 ± 0.34^b^	0.96 ± 0.08^a^	0.02 ± 0.01^a^	0.08 ± 0.00^a^	0.65 ± 0.07^a^	0.74 ± 0.07^a^
Constant	4.70 ± 0.32^a^	2.34 ± 0.13^b^	0.11 ± 0.01^b^	0.25 ± 0.03^b^	1.86 ± 0.11^b^	2.21 ± 0.13^b^
Spike	5.24 ± 0.39^b^	2.27 ± 0.23^b^	0.11 ± 0.00^b^	0.29 ± 0.03^b^	1.73 ± 0.21^b^	2.12 ± 0.20^b^
*p*-Value	0.001	0.001	0.001	0.001	0.001	0.001
**Perirenal Adipose** **(mg/g Perirenal)**						
Control	4.6 ± 0.7^a^	0.3 ± 0.0^a^	n.d	0.01 ± 0.01^a^	0.01 ± 0.00^a^	0.03 ± 0.01^a^
Constant	7.7 ± 2.6^a^	0.7 ± 0.2^b^	0.06 ± 0.02^a^	0.12 ± 0.06^b^	0.09 ± 0.03^b^	0.28 ± 0.09^b^
Spike	7.1 ± 0.6^a^	0.7 ± 0.1^b^	0.07 ± 0.01^a^	0.16 ± 0.03^b^	0.11 ± 0.04^b^	0.34 ± 0.06^b^
*p*-Value	0.543	0.001	0.001	0.001	0.001	0.001
**Muscle** **(mg/g Muscle)**						
Control	3.37 ± 0.70^b^	0.92 ± 0.38^a^	0.08 ± 0.05^a^	0.09 ± 0.01^a^	0.34 ± 0.13^a^	0.51 ± 0.19^a^
Constant	2.37 ± 0.30^a^	1.36 ± 0.14^b^	0.14 ± 0.04^b^	0.15 ± 0.02^b^	0.92 ± 0.10^b^	1.22 ± 0.15^b^
Spike	2.91 ± 0.69^b^	1.47 ± 0.06^b^	0.13 ± 0.02^b^	0.21 ± 0.02^c^	0.95 ± 0.04^b^	1.30 ± 0.05^b^
*p*-Value	0.036	0.003	0.050	0.001	0.001	0.001

Values are expressed as the mean ± SD of 6 cages per group; ^a, b, c^ Values with different superscript letters in each column differ significantly (*p* < 0.05); n.d = not detected.

Supplementation with *n*-3 LC-PUFA diets (both Constant and Spike treatments) led to a significant increase (*p* < 0.005) in whole body EPA + DPA + DHAconcentrations compared with Control treatment and the baseline data, by a factor of 50% increase, over the six weeks of the experiment (data not shown).

In terms of tissue concentrations, no significant differences were observed in EPA and DHA concentration between these two feeding strategies in any of these four tissues. In contrast, rats on the Spike treatment had significantly higher concentrations of DPA than those on the Constant treatment in liver and muscle tissue, but not in heart or adipose tissue (Table 6).

Although EPA was the main *n*-3 LC-PUFA in the Constant and Spike diets, DHA was the predominant *n*-3 LC-PUFA in all tissues analysed except perirenal fat. DPA was the next most deposited *n*-3 LC-PUFA in tissues, except the in liver, where EPA levels were significantly higher than DPA levels.

### 3.6. EPA, DPA and DHA—Mass Balance

*Intake and excretion*: The Constant treatment provided significantly more EPA, DPA and DHA to rats compared with the Spike treatment over the duration of the experiment, as noted earlier. Rats under the Constant treatment excreted significantly more DPA (*p* < 0.001) and less EPA (*p* < 0.001) and DHA (*p* < 0.001) compared with those in the Spike treatment. In addition, *n*-6 PUFAintake and excretion were higher in rats in the Constant compared with the Spike treatment.

*Accumulation*: The total *n*-3 LC-PUFA content in whole body in both *n*-3 enriched treatments over the 6 weeks of the experiment was significantly higher compared with the Control treatment (*p* < 0.05), with no effect between the two feeding strategies being observed (Table 7).

*Appearance/disappearance*: There was a net disappearance of all *n*-3 LC-PUFA over the duration of the experiment (intake greater than accumulation) in both Spike and Constant treatments; with the exception of the Control treatment where a small net appearance was observed, due to formation from the dietary 18:3n-3 (Table 7). A statistically significant difference in the net disappearance of all *n*-3 LC-PUFA were observed as a result of dietary treatment, with values for the Constant treatment being higher than for the Spike treatment (*p* < 0.05).

**Table 7:** Selected fatty acids and fatty acid classes balance (mg/animal) in rats fed under the different dietary treatments.

	PUFA *n*-6		PUFA *n*-3		EPA		DPA		DHA		EPA + DPA +DHA	
**Total Intake (mg/Animal)**												
Control	12812 ± 0 ^a^		807 ± 0 ^a^		n.d		n.d		n.d		n.d	
Constant	14685 ± 84 ^c^		4256 ± 24 ^c^		1662 ± 10 ^b^		237 ± 1 ^b^		1104 ± 6 ^b^		3003 ± 17 ^b^	
Spike	13115 ± 9 ^b^		3161 ± 10 ^b^		1208 ± 5 ^a^		145 ± 1 ^a^		779 ± 3 ^a^		2132 ± 9 ^a^	
*p*-Value	0.001		0.001		0.001		0.001		0.001		0.001	
**Excretion (mg/Animal)**												
Control	84 ± 0 ^a^		4 ± 0 ^a^		n.d		n.d		n.d		n.d	
Constant	64 ± 0 ^b^		46 ± 0 ^c^		10 ± 0 ^a^		8 ± 0 ^b^		13 ± 0 ^a^		31 ± 0 ^a^	
Spike	84 ± 0 ^a^		45 ± 0 ^b^		12 ± 0 ^b^		5 ± 0 ^a^		14 ± 0 ^b^		31 ± 0 ^b^	
*p*-Value	0.001		0.001		0.001		0.001		0.001		0.001	
**Net Intake (mg/Animal)**												
Control	12728 ± 0 ^a^		807 ± 0 ^a^		n.d		n.d		n.d		n.d	
Constant	14621 ± 84 ^c^		4210 ± 24 ^c^		1652 ± 9 ^b^		230 ± 1 ^b^		1090 ± 6 ^b^		2972 ± 17 ^b^	
Spike	13031 ± 9 ^b^		3117 ± 10 ^b^		1196 ± 5 ^a^		140 ± 1 ^a^		764 ± 3 ^a^		2101 ± 9 ^a^	
*p*-Value	0.001		0.001		0.001		0.001		0.001		0.001	
**Initial Body Content (mg/Animal)**												
Control	1331 ± 79 ^a^		167 ± 10 ^a^		4 ± 0 ^a^		18 ± 1 ^a^		44 ± 3 ^a^		66 ± 4 ^a^	
Constant	1292 ± 107 ^a^		162 ± 13 ^a^		3 ± 0 ^a^		18 ± 1 ^a^		43 ± 4 ^a^		64 ± 5 ^a^	
Spike	1274 ± 75 ^a^		160 ± 9 ^a^		3 ± 0 ^a^		18 ± 1 ^a^		42 ± 3 ^a^		63 ± 4 ^a^	
*p*-Value	0.236		0.127		0.118		0.090		0.417		0.236	
**Final Body Content (mg/Animal)**												
Control		5183 ± 1214 ^a^		386 ± 108 ^a^		3 ± 3 ^a^		28 ± 9 ^a^		144 ± 67 ^a^		175 ± 76 ^a^
Constant		4450 ± 384^a^		723 ± 28 ^b^		66 ± 25 ^b^		89 ± 30 ^b^		360 ± 27 ^b^		616 ± 36 ^b^
Spike		4930 ± 1042 ^a^		801 ± 204 ^b^		67 ± 15 ^b^		100 ± 21 ^b^		416 ± 133 ^b^		582 ± 168 ^b^
*p*-Value		0.346		0.001		0.001		0.001		0.001		0.001
**Accumulation (mg/Animal)**												
Control		3852 ± 1285 ^a^		219 ± 117 ^a^		n.d		9.5 ± 10 ^a^		100.2 ± 69 ^a^		109 ± 80 ^a^
Constant		3158 ± 227 ^a^		561 ± 26 ^b^		63 ± 9 ^a^		71 ± 9 ^b^		317 ± 28 ^b^		452 ± 38 ^b^
Spike		3656 ± 1045 ^a^		641 ± 204 ^b^		63 ± 15 ^a^		82 ± 21 ^b^		373 ± 133 ^b^		519 ± 168 ^b^
*p*-Value		0.476		0.001		0.001		0.001		0.001		0.001
**Appearance/Disappearance (mg/Animal)**												
Control		−8876 ± 1285 ^a^		−584 ± 117 ^a^		0.3 ± 3 ^a^		10 ± 10 ^a^		100 ± 69 ^a^		109 ± 80 ^a^
Constant		−11463 ± 441 ^a^		−3649 ± 34 ^c^		−1589 ± 13 ^c^		−158 ± 9 ^c^		−772 ± 23 ^c^		−2520 ± 30 ^c^
Spike		−9375 ± 1043 ^a^		−2475 ± 198 ^b^		−1133 ± 13 ^b^		−58 ± 21 ^b^		−391 ± 131 ^b^		−1582 ± 162 ^b^
*p*-Value		0.871		0.001		0.001		0.001		0.001		0.001

Values are expressed as the mean ± SD of 6 cages per group; ^a, b, c^ Values with different superscript letters differ significantly in each column (*p* < 0.05); n.d = not detected; PUFA (polyunsaturated fatty acid) n-6: 18:2n-6, 20:2n-6, 22:2n-6, 18:3n-6, 20:3n-6, 20:4n-6, 22:4n-6, 24:4n-6, 24:5n-6, 22:5n-6; PUFA *n*-3: 18:3n-3, 20:3n-3, 22, 3n-3, 18:4n-3, 20:4n-3, 20:5n-3, 22:5n-3, 24:5n-3, 24:6n-3, 22:6n-3.

### 3.7. EPA, DPA and DHA Apparentin Vivo Metabolism

The Whole Body Fatty Acid Balance Method allowed the calculation of apparent *in vivo* fatty acid metabolism (excretion, β-oxidation, bioconversion and deposition) as shown in Table 8. The two most important pathways identified for the *n*-3 LC-PUFA in this study were β-oxidation and deposition, which together accounted for more than 98% of the apparent *in vivo* metabolism (Table 8).

Based on calculation, the main fate of EPA, DPA and DHA in both *n*-3 LC-PUFA supplemented treatments was β-oxidation, except for DPA in rats fed the Spike treatment where deposition was observed as the main fate. β-oxidation was approximately 95% for EPA, while for DPA and DHA it ranged between 40% to 70%. There was a significant effect of the dietary treatment on the β-oxidation for EPA, DPA and DHA, which was higher for rats fed the Constant compared with Spike treatment.

Apart from β-oxidation, deposition was the next main fate of the EPA and DHA in both treatments and DPA in the Constant treatment. In the case of EPA, deposition amounted to less than 6% of the total metabolic activity, whereas in the case of DPA and DHA, deposition accounted for between 30% to 56% and 28% to 48%, respectively, depending on treatment (Table 8). The whole body deposition of all *n*-3 LC-PUFA was significantly higher (*p* < 0.05) in rats fed the Spike treatment compared with those fed the Constant treatment.

For EPA and DHA there were very similar trends between Spike and Constant treatments (for both treatments: β-oxidation > deposition > excretion); however, this was not the case for DPA since for the Constant treatment β-oxidation > deposition > excretion, while for the Spike treatment deposition > β-oxidation > excretion. DPA was likely not further bio-converted to other *n*-3 LC-PUFA (namely 24:5n-3, 24:6n-3 or DHA) in any treatment. In the control group, 18:3n-3 was bioconverted at a significantly higher rate compared with the other two treatments.

The *n*-6 fatty acid β-oxidation, bioconversion and deposition were similar between all three dietary treatments. However, the n-6 fatty acid excretion was significantly higher (*p* < 0.001) in the Spike compared with the Constant treatment. In the Constant and Spike treatments mostly 18:3n-6 and 20:4n-6 were bioconverted, 20:3n-6 was bioconverted only in the Spike treatment. In the Constant treatment, mostly 18:2n-6 and 20:4n-6 were deposited while in the Spike treatment, more n-6 fatty acids (18:2n-6, 20:4n-6, 22:4n-6, 22:5n-6) were deposited in the rat’s whole body (data not shown).

**Table 8:** Metabolic fate of fatty acid (% of the intake) in rats under the three dietary treatments.

	PUFA *n*-6	PUFA *n*-3	EPA	DPA	DHA	EPA + DPA + DHA
**Excreted (% of Intake)**						
Control	0.65 ± 0.00 ^c^	0.52 ± 0.00 ^a^	n.d	n.d	n.d	n.d
Constant	0.43 ± 0.00 ^a^	1.07 ± 0.00 ^b^	0.57 ± 0.00 ^a^	3.18 ± 1.01 ^a^	1.23 ± 0.50 ^a^	1.00 ± 0.00 ^a^
Spike	0.62 ± 0.00 ^b^	1.40 ± 0.00 ^c^	1.02 ± 0.00 ^b^	3.52 ± 0.00 ^b^	1.84 ± 0.00 ^b^	1.44 ± 0.00 ^b^
*p*-Value	0.001	0.001	0.001	0.001	0.001	0.001
**β-Oxidised (% of Intake)**						
Control	69.78 ± 9.75 ^a^	74.81 ± 12.84 ^a^	n.d	n.d	n.d	n.d
Constant	78.23 ± 2.74 ^a^	86.30 ± 0.74 ^a^	95.66 ± 0.57 ^b^	66.61 ± 3.62 ^b^	69.98 ± 2.43 ^b^	84.44 ± 1.20 ^b^
Spike	71.80 ± 7.83 ^a^	79.29 ± 6.11 ^a^	93.78 ± 1.24 ^a^	40.00 ± 14.60 ^a^	50.21 ± 17.01 ^a^	75.14 ± 7.49 ^a^
*p*-Value	0.096	0.081	0.012	0.008	0.037	0.001
**Bio-Converted (% of Intake)**						
Control	4.90 ± 2.81 ^b^	15.72 ± 10.10 ^b^	n.d	n.d	n.d	n.d
Constant	1.56 ± 0.08 ^a^	0.16 ± 0.02 ^a^	n.d	n.d	n.d	n.d
Spike	3.07 ± 1.60 ^a,b^	0.05 ± 0.08 ^a^	n.d	n.d	n.d	n.d
*p*-Value	0.026	0.001	-	-	-	-
**Deposited (% of Intake)**						
Control	24.67 ± 7.05 ^a^	8.95 ± 3.28 ^a^	n.d	n.d	n.d	n.d
Constant	19.78 ± 2.70 ^a^	12.47 ± 0.75 ^a^	3.76 ± 0.57 ^a^	30.21 ± 3.87 ^a^	28.79 ± 2.43 ^a^	14.51 ± 1.21 ^a^
Spike	24.51 ± 6.32 ^a^	19.26 ± 6.04 ^b^	5.24 ± 1.24 ^b^	56.50 ± 14.60 ^b^	47.9 ± 17.0 ^b^	23.42 ± 7.49 ^a^
*p*-Value	0.269	0.002	0.033	0.008	0.042	0.034

Values are expressed as the mean ± SD of 6 cages per group. ^a, b, c^ Values with different superscript letters differ significantly in each column (*p* < 0.05); n.d = not detected; PUFA (polyunsaturated fatty acid) n-6: 18:2n-6, 20:2n-6, 22:2n-6, 18:3n-6, 20:3n-6, 20:4n-6, 22:4n-6, 24:4n-6, 24:5n-6, 22:5n-6; PUFA n-3: 18:3n-3, 20:3n-3, 22:3n-3, 18:4n-3, 20:4n-3, 20:5n-3, 22:5n-3, 24:5n-3, 24:6n-3, 22:6n-3.

The apparent *in vivo* enzyme activities (expressed as mmol of product per g of body weight per day) are reported in Table 9. The apparent total elongase activity and desaturase activities (Δ-5 Desaturase and Δ-6 Desaturase) were similar in rats under the Constant and the Spike treatment. The apparent Δ-9 Desaturase enzyme activity was higher in rats under the Spike treatment compared with those on the Constant treatment.

**Table 9:** Apparent *in vivo* enzyme activities in rats fed the three dietary treatments.

	Ƹ Elongase	Ƹ Δ-9 Desaturase	Ƹ Δ-6 Desaturase	Δ-6 Desaturase for n-6	Δ-6 Desaturase for n-3	Ƹ Δ-5 Desaturase	Δ-5 Desaturase for n-6	Δ-5 Desaturase for n-3
Control	629 ± 335 ^b^	1305 ± 245 ^c^	298 ± 192 ^b^	228 ± 142 ^b^	69 ± 49	227 ± 147 ^b^	195 ±122 ^b^	31 ± 24
Constant	97 ± 15 ^a^	375 ± 134 ^a^	48 ± 4 ^a^	48 ± 4 ^a^	n.d	56 ± 5 ^a^	56 ± 5 ^a^	n.d
Spike	219 ± 146 ^a^	722 ± 240 ^b^	114 ± 67 ^a,b^	114 ± 67 ^a,b^	n.d	108 ± 61 ^a,b^	108 ±61 ^a,b^	n.d
*p*-Value	0.001	0.001	0.006	0.012	-	0.017	0.025	-

Values are expressed in mmol/g/day as the mean ± SD of 6 cages per group; ^a, b, c^ Values with different superscript superscript letters differ significantly in each column (*p* < 0.05); n.d = not detected. The Greek letter capital sigma (Σ) indicates summation.

## 4. Discussion

The present study sought to examine the whole body bioavailability and efficiency (as *n*-3 LC-PUFA metabolic fate) of the same overall dose of *n*-3 LC-PUFA, provided from the same source (fish oil), at the same overall weekly dose, but at different frequencies: daily*vs.* weekly. It was found that the growth of the animals was not different between treatments and no mortalities were recorded amongst the three experimental treatments (Constant, Spike and Control). There were significant but small differences in the excretion of the *n*-3 LC-PUFA between the Spike and Constant treatment groups, which are unlikely to be nutritionally relevant.

The results obtained by simply comparing tissue FA concentrations (mg/g tissue) were interesting, but they are admittedly of limited value towards achieving a better understanding of *n*-3 LC-PUFA “bioavailability”. While the dietary intake of *n*-3 LC-PUFA provided by the two diets were similar, but not identical, there were some differences between treatments that might be independent of the difference in dietary intake. For example, the DPA concentration in liver and muscle was significantly greater in the Spike treatment than in the Constant treatment, despite the dietary DPA intake being significantly greater for the Constant treatment. This has no obvious explanation, but reveals that metabolic processing of dietary *n*-3 LC-PUFA is more complex than simply looking at dietary intake values or tissue levels.

A much greater and more accurate understanding of the actual metabolic fate of *n*-3 LC-PUFA provided by different oils can be achieved by observing the results of the Whole Body Fatty Acid Balance Method, which takes into account, and thus balances out, any differences in dietary intake.

This data showed that the major metabolic fate of the *n*-3 LC-PUFA (EPA + DPA + DHA) was towards catabolism (β-oxidation) accounting for over 70% of total dietary intake, whereas deposition accounted less than 25% of total dietary intake. It was found that significantly more *n*-3 LC-PUFA were β-oxidised when originating from the Constant treatment (84% of dose), compared with the Spike treatment (75% of dose). Conversely, it was found that significantly more *n*-3 LC-PUFA were deposited when originating from the Spike treatment (23% of dose), than from the Constant treatment (15% of dose). This result suggests that the *n*-3 LC-PUFA provided by the Spike treatment were more deposited (bioavailable), compared with those provided by the Constant treatment.

The differences in β-oxidation and deposition were not the same for each of EPA, DPA and DHA for either the Spike or Constant treatment. That is, EPA was more extensively β-oxidised than DPA and DHA on both treatments, but the differences between the 20 carbonand the 22 carbon PUFA were accentuated in the Spike treatment. In the case of β-oxidation, the Constant/Spike ratios were 1.7 for DPA and 1.4 for DHA, but only 1.0 for EPA. In terms of deposition, the Constant/Spike ratios were 0.5 for DPA, 0.6 for DHA, and 0.7 for EPA. This suggests that EPA is preferentially directed towards β-oxidation almost independent of whether the EPA is provided daily or once per week. This is consistent with data showing high affinity of EPA to catabolism (β-oxidation) in animal models [23]. It has been reported using Wistar rats that EPA-CoA was a good substrate for mitochondrial carnitine acyl-transferase-I and DHA was a poor substrate for both mitochondrial and peroxisomal β-oxidation, which could explain the high rate of β-oxidation for EPA [23].

In contrast to EPA, it would appear that DHA and DPA are somewhat spared from β-oxidation when consumed, as observed previously [24] but especially when a large dose of dietary *n*-3 LC-PUFA is provided weekly. This is consistent with the finding of Kaur *et al.* [24] in rodents who showed, using radiolabelled EPA, DPA and DHA in rats, that six hours after dosing 19% of the EPA was β-oxidized and expired as CO_2_ compared with 5% in case of DPA and 7% of DHA. However, these data do not shed any light on why providing a bolus dose of *n*-3 LC-PUFA leads to a greater partitioning of DPA and DHA towards deposition. With the Spike treatment, it is therefore possible to speculate that this “flood” of *n*-3 LC-PUFA could have saturated the capacity of the mitochondria to β-oxidise any extra DPA and DHA, resulting in a greater retention and deposition of these fatty acids in tissues.

To our knowledge, this is the first study to compare the whole body bioavailability of *n*-3 LC-PUFA in rats fed a constant daily dose*vs.* a larger and less frequent dose of the same dietary source of *n*-3 LC-PUFA. From the two available human studies comparing a weekly dose of *n*-3 LC-PUFA with daily dose, only one study used the same source of *n*-3 LC-PUFA (capsules not fish meal) [12]. Both studies used blood levels (plasma, platelets or mononuclear cells) as a proxy for bioavailability. The limitations of these studies include a failure to provide the dose adjusted on a body weight basis, the failure to measure excretion and the failure to measure the EPA and DHA levels in the red blood cells (which are widely regarded as the best measure of EPA + DHA tissue status). Furthermore, because of the known high level of variability in the response of subjects to the same dose of fish oil (as noted by Kohler *et al.* [25]), these studies have limitations because the data was not adjusted for by gender, body weight or exercise level [26]. In the present rat study, rats were fed to fixed predetermined ration, which was adjusted weekly relative to body weight, for 6 weeks which helped to reduce the variability of the results achieved, increased the statistical power of the test (greater than 80% for the vast majority of data recorded), and ultimately contributed to obtaining more robust, substantiated and more easily interpretable findings.

The possible difference in bioavailability of *n*-3 LC-PUFA when provided in different edible sources has received some research attention. Specifically, the blood levels of *n*-3 LC-PUFA derived from daily fish oil capsules compared with either adaily fish meal or daily fish oil enriched food have been reported in a few studies [27,28,29,30].

In the current study, the Constant and the Spike treatment showed lower levels of apparent *in vivo* enzyme activity for elongase, Δ6 and Δ5 desaturase compared with the Control treatment. Lower Δ6 desaturase activity with fish oil feeding has been previously reported [31]; however, there is no data looking at a weekly*vs.* daily dose to compare with. These desaturases and elongases are required for the biosynthesis of LC-PUFA and their inter-conversion. High availability of these fatty acids in Constant and the Spike treatment can act via a negative feedback control mechanism and reduce the gene transcription rate and the actual activity of the desaturase and elongase enzymes in these treatments, possibly via sterol regulatory element binding protein (SREBP-1c) [32,33]. On the other hand, the lack of *n*-3 LC-PUFA intake in the diet of the Control rats has likely increased their elongase and desaturase enzyme activities in order to increase endogenous *n*-3 LC-PUFA synthesis. Dietary *n*-3 LC-PUFA deprivation has previously been shown to upregulate liver mRNA levels of Δ6 and Δ5 desaturases as well as activities of Δ6 and Δ5 desaturases [34,35].

Overall, there were no significant differences in the assessed enzyme activities between the Spike and Constant treatments, with the exception of Δ9 desaturase (required for the biosynthesis of monounsaturated fatty acids), which was significantly higher in the Spike treatment.

Admittedly, one of the limitations of the present study includes being an animal study in male rats. Nevertheless, these preliminary, novel and highly interesting findings warrant further investigations, and in particular the need to be substantiated by conducting appropriate trials in humans. It is worth noting that the dose of *n*-3 LC-PUFA used in the Constant treatment equates to 1012 mg/day for a 70 kg human [36], which is in the range of human recommendations for these fatty acids. Another limitation, as previously mentioned, was that the slightly different total amounts of *n*-3 LC-PUFA administered by the two *n*-3 LC-PUFA enriched dietary treatments used in this study. However, these differences are relatively minimal, and unlikely to be responsible of any major modification in the overall *n*-3 LC-PUFA metabolism, and have been accounted for in the Whole Body Fatty Acid Balance Method. A third limitation is that these results do not apply to comparative effects of consumption of daily fish oil capsules *vs.* a sporadic meal with fish or seafood, since the study investigated the bioavailability of the same food source of *n*-3 LC-PUFA; but this was intentional to exclude the possible effect of the matrix (food source) of the dietary *n*-3 LC-PUFA.

In conclusion, our data show that there was a significantly greater deposition of the *n*-3 LC-PUFA associated with a single large dose of dietary *n*-3 LC-PUFA compared with the smaller daily doses in rats, due to less β-oxidation and greater deposition, and not due to differences in excretion (digestibility). The results from this animal study provide a suitable platform for future human studies aimed at developing substantiated evidence for advising consumers on the most efficient way to increase their *n*-3 LC-PUFA status.These findings suggests that a large dose of *n*-3 LC-PUFA once per week is more effective in increasing whole body *n*-3 LC-PUFA content compared to a smaller dose delivered daily. This observation, if validated in humans, could have remarkable effects on the possible development of more effective and sustainable utilisation strategies of these limited and metabolically important nutrients, currently derived primarily from the dwindling oceanic fish stocks.
